# A Study on the Immunoregulatory Role of the PD1 Pathway in Juvenile Idiopathic Arthritis

**DOI:** 10.31138/mjr.140523.aso

**Published:** 2023-08-29

**Authors:** Artemis Koutsonikoli, Anna Taparkou, Polyxeni Pratsidou-Gertsi, Vasiliki Sgouropoulou, Maria Trachana

**Affiliations:** First Department of Paediatrics, Hippokration General Hospital, Aristotle University of Thessaloniki, Thessaloniki, Greece

**Keywords:** PD1 pathway, JIA, immune checkpoint, biomarkers

## Abstract

**Objectives:**

To investigate the immunoregulatory role of the Programmed-cell-Death-protein-1 (PD1) pathway, an inhibitory immune checkpoint, in Juvenile Idiopathic Arthritis (JIA).

**Methods:**

The PD1 expression on CD4+ and CD8+ T-cells was determined by flow cytometry and the PD1 soluble form (sPD1) levels by ELISA, in peripheral blood (PB)/serum and synovial fluid (SF) samples of JIA patients and healthy controls (HCs). We searched for any association in-between the biomarkers and with JIA activity.

**Results:**

101 Caucasian patients (69 female), aged 12 (8–15) years, and 20 HCs participated in this study. The PB PD1 expression on T-cells was higher in: a. JIA patients *vs* HCs (CD4: 1.24% *vs* 0.32%, p=0.007, CD8: 1.6% *vs* 0.4%, p=0.002). b. active *vs* inactive JIA (CD4: 1.44% *vs* 0.87%, p=0.072, CD8: 2.1% *vs* 0.93%, p=0.005). The SF PD1 expression on T-cells correlated strongly and positively with the disease activity (CD4: ρ=0.55, p=0.022, CD8: ρ=0.555, p=0.026). The SF PD1 expression on CD8 T-cells was higher in patients on-treatment *vs* those off-treatment (21.3% *vs* 5.83% p=0.004). The sPD1 levels were higher in the SF *vs* the serum (801pg/ml *vs* 367.2, p=0.013), without an association with disease activity.

**Conclusion:**

These results indicate an up-regulation of the PD1-pathway in JIA, at least quantitatively, especially in active disease. sPD1 is compartmentally produced at the inflamed joints. Further investigation in a larger sample of JIA patients may verify these observations and contribute to unravelling the precise role of the PD1 pathway in the pathogenesis and persistence of the joint inflammation.

## INTRODUCTION

Juvenile Idiopathic Arthritis (JIA) is the most common paediatric rheumatic disease, affecting approximately 32.6 per 100000 Caucasian children and adolescents.^1^ It is a chronic disease with unpredictable course, characterised by periods of flare and remission.^2^ About 50% of children with JIA continue to have symptoms as adults.^2^ Once a disease with serious complications, nowadays its prognosis has improved, mainly due to the use of biologic therapies.^3,4^ Early diagnosis and aggressive treatment are major contributors to a favourable outcome.^2,4–6^

JIA is an autoimmune disease, with multiple genetic and environmental factors involved in the pathogenesis. Defects and imbalances in both cell-mediated and humoral immunity lead to the breakdown of self-tolerance and the initiation and sustainment of inflammation at the target-organs. Despite accumulated research, several aspects of the pathophysiology of JIA remain poorly elucidated, raising obstacles to the development of personalised treatment strategies.^2,7^

Among the investigated inflammatory pathways in the initiation of autoimmunity disorders are the Immune Checkpoints (ICs). They are regulators of the immune responses, critical for the prevention of excessive immune activation and the maintenance of self-tolerance.^8^ Their modulation has garnered scientific interest, especially as targets of cancer therapy.^9^ The pathway consisting of the Programmed cell Death protein-1 (PD-1) and its ligands (PDL-1 and 2) is one of the newer reported ICs. PD-1 is a transmembrane protein, expressed on T-, B- and other immune cells upon their activation. Its expression is induced and sustained in settings of persistent antigen stimulation. By the binding of PD-1 with its ligands, the pathway delivers inhibitory signals; namely, it limits T-cells’ activation, proliferation, survival and cytokine production. It also enhances the differentiation and function of T-regulatory cells (Tregs). These properties have rendered the PD-1 pathway essential for the prevention of autoimmunity, as it can thwart auto-reactive T-cells.^10^ The soluble isoform of PD-1 (sPD-1) may antagonize the membrane-bound PD-1, by binding to its ligands, thus inhibiting the pathway’s effects.^10^

Based on the abovementioned observations, this study’s hypothesis was that the PD-1 pathway might dysfunction in young patients with autoimmune disorders. Since relevant data in JIA are still limited, we aimed to investigate the PD-1 pathway’s activity in JIA patients, during disease activity and remission. Our primary objectives were the determination of the: a. serum and synovial fluid (SF) levels of sPD-1 and b. PD-1 expression on T-helper (CD4) and T-cytotoxic (CD8) cells in the peripheral blood (PB) and SF. The secondary objectives were the investigation of the interplay between the above biomarkers, as well as their association with JIA activity.

## METHODS

This was a case-control study including JIA patients and healthy controls (HCs).

### Patients’ inclusion criteria

a. Fulfilment of the ILAR (International League of Associations for Rheumatology) classification criteria and b. follow-up at the study Centre, where a large number of patients with rheumatic and autoimmune diseases from different regions are monitored.^11^ The HCs group consisted of children, age- and gender-matched to the patients*,* without evidence of an autoimmune or recent inflammatory disorder. Prior to inclusion in the study, an informed consent from each participant/parent was received, according to the Helsinki Declaration.

### Patient characteristics

The demographic data of all study participants were recorded. Clinical, laboratory findings and current treatment at the time of sample collection were also recorded in JIA patients. JIA subtypes were classified according to the established ILAR criteria.^11^ Patients who did not meet the definition for inactive disease, according to Wallace criteria, were defined as having active disease.^12^ Disease activity was further quantified using the Juvenile Arthritis Disease Activity Score-10 (JADAS-10) tool.^13^

### Biosamples

From each study participant, an additional peripheral blood sample was drawn for the purposes of the study, at the time of a necessary laboratory evaluation. From some patients in active JIA, a second blood sample was collected at the time of remission and at least 3 months after the initial sample. SF samples were collected from patients who underwent a therapeutic intra-articular corticosteroid injection. For the determination of sPD-1, we also used previously stored serum and SF samples from our biobank.

Each blood and synovial fluid sample was divided. One portion was used for the immediate identification of cell populations, while the remainder was centrifuged and stored at −80°C for the subsequent determination of soluble biomarkers.

### Flow Cytometry

The investigation of the PD-1 expression on CD4 and CD8 T-cells was performed by multiparametric-polychromatic flow cytometry (quintuple fluorescence protocol). The following monoclonal antibodies bound to the appropriate fluorochromes were used: CD3 FITC, CD4 PE-Cy7, CD8 APC-Cy7, CD45 PerCP-Cy5.5, CD279 PE and the appropriate isotype matched control antibody PE to determine PD1.

All monoclonal antibodies were purchased from EXBIO (Praha Czech Republic).

Sample preparation was performed according to standard protocols and acquisition of 50.000 events was performed within 30 minutes on MINDRAY flow cytometry analyser. Lymphocytes were identified by their forward (FSC) and side scatter (SSC) characteristics combined with the CD45 expression, whereas T-cells, T-helper (CD4) and T-cytotoxic (CD8) cells were identified by the expression of the specific markers anti-CD3, anti-CD4 and anti-CD8, respectively.

### Determination of sPD1 levels

Commercially available enzyme-linked immunosorbent assay (ELISA) was used to determine sPD1 (Abcam, Cambridge, UK).

All the analyses were performed at the Immunology laboratory of the Centre.

### Statistical analysis

The Shapiro-Wilk test was used to test the normality of continuous variables. Since most of the continuous variables were non-normally distributed, results were reported using the median value and quartiles (Q1–Q3). Categorical variables were reported using percentages and in-between group differences were tested using the chi-square test. For the comparison of paired continuous variables, the Wilcoxon-signed rank test was used, while in-between group differences for independent continuous variables were tested using the Mann-Whitney test. In case of more than 2 groups the Kruskal-Wallis test was used. Spearman’s coefficient was calculated to test for correlations between two continuous variables. A p-value <0.05 was considered as statistically significant. The statistical analysis was conducted using the statistical package IBM SPSS statistics version 27.0.

## RESULTS

### Participants’ and samples’ characteristics

Overall, one hundred and one JIA patients participated in this study. The demographic and clinical characteristics of the study’s participants are shown in **Table 1**. Twenty healthy children served as controls.

**Table 1.** The demographic and clinical characteristics of the study’s participants.

	**JIA patients**	**Healthy controls**	**p**

**Number of participants**	101	20	

**Female (%)**	68	60	ns

Age (years)*	12 (8–15)	13 (10.5–14.1)	ns

Disease duration (years)*	3 (1–8)	-	-

Disease duration post-diagnosis (years)*	2 (0–7.3)	-	-

**JIA subtype (%)**			
**-Polyarthritis**	28.1		
**-Oligoarthritis**	32.3		
**-Psoriatic**	11.5	-	-
**-ERA**	16.7		
**-Systemic**	8.3		
**-Unclassified**	3.1		

**ANA positive (%)**	51	-	-

**No of serum samples for sPD1 measurement**			
**- total**	81	20	-
**- in active disease**	55	-	

**No of PB samples for PD1 cellular expression**			
**- total**	56	20	-
**- in active disease**	39	-	

**No of SF samples for sPD1 measurement**	36	-	-

**No of SF samples for PD1 cellular expression**	28	-	-

* median (Q1–Q3); ERA: enthesitis-related arthritis; JIA: Juvenile Idiopathic Arthritis; ns: not significant; PB: peripheral blood; PD: programmed cell death; sPD1: soluble PD1; SF: synovial fluid.

### Comparison of the biomarkers in patients vs healthy controls

**Figure 1.**
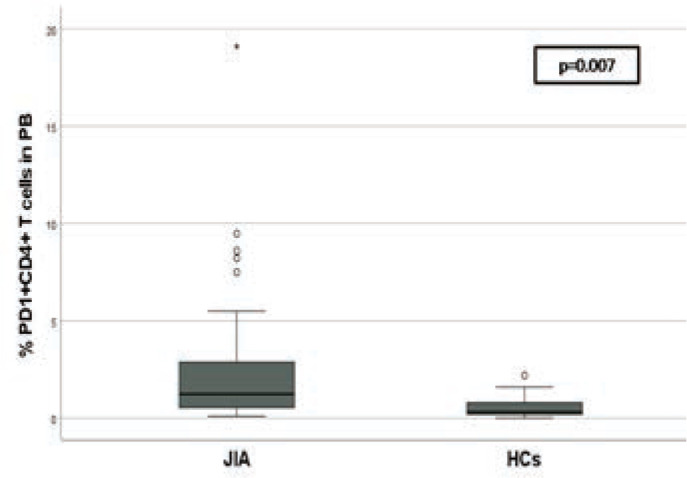
Percentage of PD1+CD4+ T-cells in the peripheral blood (PB) of JIA patients and Healthy Controls (HCs).

**Figure 2.**
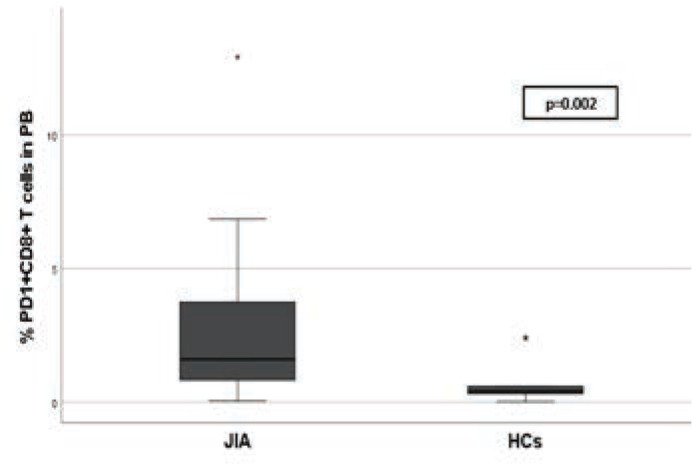
Percentage of PD1+CD8+ T-cells in the peripheral blood (PB) of JIA patients and Healthy Controls (HCs).

Patients with JIA had a significantly higher PB PD1 expression on both CD4 and CD8 T-cells compared to HCs (CD4: 1.24% Q1–Q3: 0.54–2.9 *vs* 0.32% Q1–Q3: 0.17–1, p=0.007, CD8: 1.6% Q1–Q3: 0.81–3.8 *vs* 0.4% Q1–Q3: 0.26–1.04, p=0.002, **Figures 1 and 2**). However, the serum sPD1 levels did not differ significantly between patients and HCs.

### Association of the biomarkers with JIA activity

#### A. PB findings

**Figure 3.**
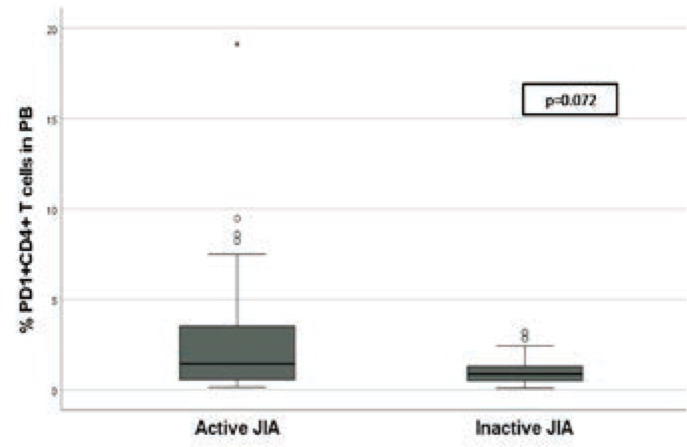
Percentage of PD1+CD4+ T-cells in the peripheral blood (PB) of JIA patients with active and inactive disease.

**Figure 4.**
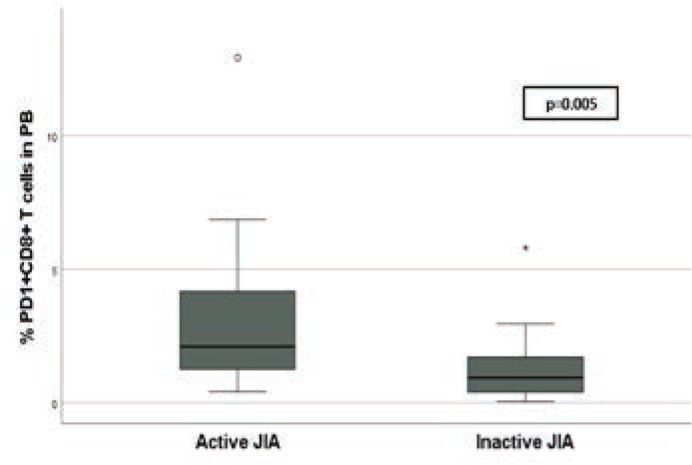
Percentage of PD1+CD8+ T-cells in the peripheral blood (PB) of JIA patients with active and inactive disease.

- Patients with active disease had a higher PD1 expression on both CD4 and CD8 T-cells, as compared to patients with inactive disease. (CD4: 1.44% Q1–Q3: 0.55–3.65 *vs* 0.87% Q1–Q3: 0.45–1.78, p=0.072, CD8: 2.1% Q1–Q3: 1.23–4.2 *vs* 0.93% Q1–Q3: 0.38–1.79, p=0.005, **Figures 3 and 4**).
- PD1 expression on CD4 T-cells correlated moderately and positively with the erythrocyte sedimentation rate (ESR) (ρ=0.311, p=0.025).
- Regarding CD8 T-cells, their PD1 expression correlated moderately and positively with the MD-VAS (Physician’s global assessment on a visual analogue scale) (ρ=0.315, p=0.019), the number of affected joints (ρ=0.337, p=0.012) and the JADAS-10 (ρ=0.323, p=0.021).
- In 3 patients with active disease the PB PD1 expression on T-cells was determined as well when they were in remission. The sample size was too small, but there was a trend towards lower PD1 expression on CD8 cells during remission.

#### B. SF findings

- The PD1 expression on CD4 T-cells correlated strongly and positively with the number of affected joints (ρ=0.55, p=0.022).
- Regarding CD8 T-cells, their PD1 expression correlated strongly and positively with the MD-VAS (ρ=0.555, p=0.026).

However, the disease activity was not found to be associated with the serum or SF sPD1 levels. Furthermore, none of the studied biomarkers differed significantly between patients at disease onset and those with a disease flare.

### Comparison of the biomarkers with disease characteristics

None of the biomarkers showed significant differences between the different JIA subtypes or between patients with positive and negative ANA. In patients with active disease, there was a trend towards higher sPD1 levels in the serum of patients with systemic JIA (sJIA) compared with all the other JIA subtypes but it did not reach statistical significance.

### Effect of treatment on the biomarkers

The SF PD1 expression on CD8 T-cells was significantly higher in patients who were on treatment compared with those off treatment. (21.3% Q1–Q3: 10.4–32.57 *vs* 5.83% Q1–Q3: 1.52–11.6 p=0.004). For the rest of the biomarkers there were no significant differences.

### Concurrent comparison of the biomarkers in blood/sera vs SF

**Figure 5.**
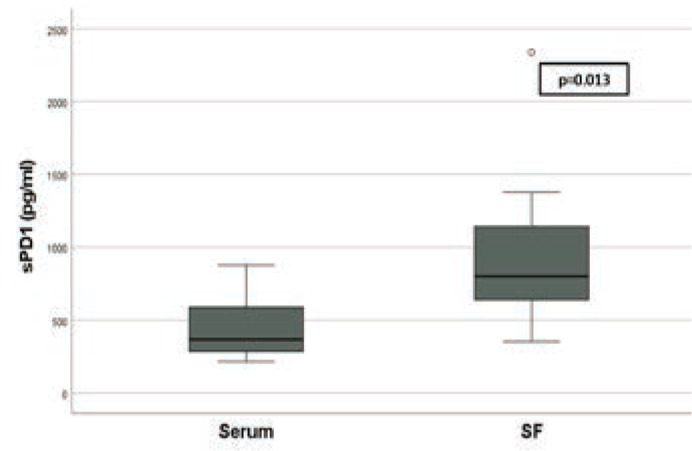
sPD1 levels in serum and synovial fluid (SF) (paired samples).

**Figure 6.**
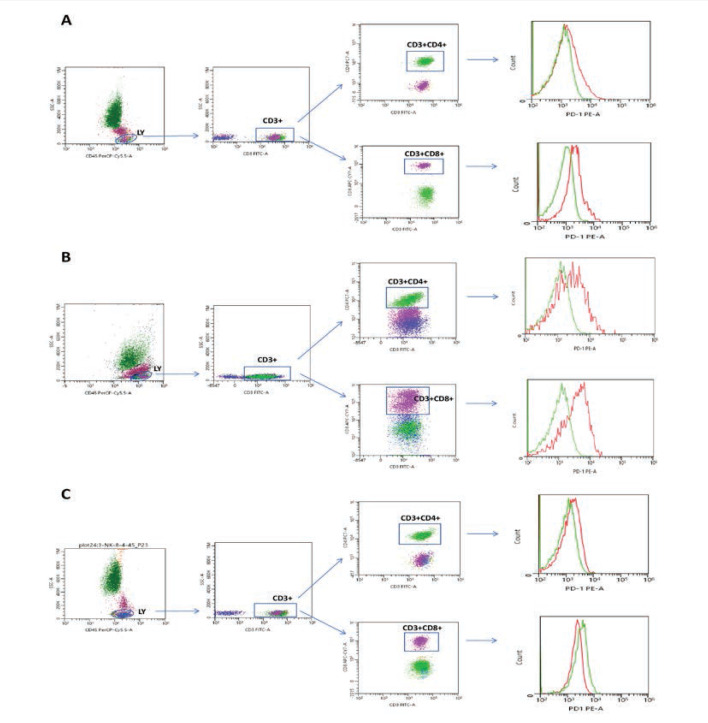
Gating strategy and PD1 expression on CD4 and CD8 T-cells. Expression of PD1 on cell subsets **(A)** in blood of a representative patient, **(B)** in synovial fluid of the same patient, **(C)** in blood of a healthy control. Green line histograms represent isotype control (unstained cells), and red line histograms represent cells stained with CD279 (PD1). The acquisition and analysis gates were restricted to the lymphocytes as determined by the forward scatter (FSC) and side scatter (SSC) characteristics of these cells combined with the CD45 expression. A CD3 vs SSC display was used to identify T-cells. T-helper and T-cytotoxic cells were identified as CD3+CD4+ and CD3+CD8+, respectively.

In 11 patients, we measured sPD1 concurrently in the serum and SF. The SF sPD1 levels were significantly higher (801pg/ml Q1–Q3: 578,4–1178 *vs* 367.2 Q1–Q3: 224.6–613.2, p=0.013, **Figure 5**). The number of patients with concurrent PB and SF samples was too low to draw conclusions, but there were no significant differences in the expression of PD1 on CD4 or CD8 cells. The expression of PD1 on each cell subset in blood and SF of a representative patient *vs* a healthy control is shown in **Figure 6**.

### Correlation between the studied biomarkers

There was a strong significant correlation between the PB PD1 expression on CD4 cells with that on CD8 cells (ρ=0.576, p<0.001), but neither correlated with the sPD1 serum levels. There was also a strong significant correlation between the SF PD1 expression on CD4 cells with that on CD8 cells (ρ=0.633, p<0.001). The SF PD1 expression on CD8 cells correlated, as well, with the SF sPD1 levels moderately and positively (ρ=0.467, p=0.012).

## DISCUSSION

This is the first study in Greece investigating the PD1 pathway in patients with JIA, and one of the few in the global literature. We determined the PD1 expression on CD4 and CD8 T-cells as well as the PD1’s soluble form (sPD1) in the PB/sera and SF of JIA patients, both in active and in inactive disease. Our aim was to gain insight into the function of the inhibitory PD-1 pathway, a presumably protective mechanism towards autoimmunity.

Our study’s findings indicate an up-regulation of the PD1-pathway, at least quantitatively. The PB PD1 expression on CD4 and CD8 T-cells was high in JIA patients, especially in active disease. There was a positive correlation of the above-mentioned biomarkers with certain indices of the disease activity, namely with the ESR, MD-VAS, number of affected joints and JADAS-10. These findings have not been previously mentioned in JIA patients. The PD1 expression on CD4 T-cells was investigated by Cai et al. in 101 Chinese JIA patients, in a study focused on revealing the diverse cell profile of sJIA. They found that in active sJIA the PD1 expression was lower compared to HCs, active polyarthritis, active ERA and inactive sJIA. There was a negative correlation of the PD1 expression with markers of disease activity in sJIA.^14^ Decreased PB expression of PDL-1 in active sJIA patients compared to other febrile illnesses has also been described in a small study, although PB PD-L1 mRNA expression was upregulated.^15^ We found no significant differences regarding the PB PD1 expression between the different subtypes of JIA. It is evident that further and larger studies in Caucasian JIA patients are needed to clarify these contradictory results.

Our results are, however, in agreement with those of Luo et al. regarding 81 Chinese RA patients, who demonstrated higher PD1 expression in PB CD4 T-cells compared to HCs, although the PD1 expression on CD8 T-cells was not significantly higher. There was also a positive correlation with markers of disease activity.^16^ Rao et al. have also detected an expanded subpopulation of positive for PD1 CD4 T-cells, namely PD1^hi^CXCR5^−^, in the PB of seropositive RA patients. These cells were higher in patients with high/moderate disease activity.^17^ Nevertheless, diminished expression of PD1 on CD4 and CD8 T-cells in RA has also been reported.^18^

Although the low number of paired PB/SF samples in the present study does not allow drawing conclusions, we observed a remarkably higher SF PD1 expression on CD4 and CD8 cells compared to PB, in unpaired samples (data not shown). The literature is more consistent regarding the site of inflammation, with multiple studies demonstrating high SF PD1 expression on T-cells in JIA and adult patients with RA.^19–27^ In our study, the SF PD1 expression positively correlated with some measures of disease activity, namely with the MD-VAS and the number of affected joints. This aligns with a previous study by Luo et al. regarding RA patients.^16^

Patients on treatment had significantly higher SF PD1 expression on CD8 T-cells compared to patients off treatment. The treatment regimens included mainly methotrexate, alone or in combination with an anti-TNF. The sample size did not allow comparison of the different treatment regimens. Since all patients had active disease at the time of SF collection, we hypothesised that either the immunomodulatory agents directly influence the PD1 pathway or the inflammatory microenvironment is altered during a disease flare while the patient is under treatment. In vitro, Bommarito et al have shown that TNF-α counteracts the effect of the PD1-pathway on T-cells, possibly by inducing sPD1 production, and the addition of an anti-TNF agent reverses this counteraction.^26^ However, the sPD1 levels did not differ significantly in the aforementioned samples of our study.

Despite the up-regulated expression of PD1 in the periphery and especially at the site of inflammation, the pathway seems unable to inhibit the inflammatory process that characterises JIA. One proposed mechanism is the presence of sPD1 which blocks the PD1 pathway by antagonistically binding to the membrane-bound PD1’s ligands.^10^ This hypothesis is supported by the majority of studies in RA, which have demonstrated high serum and SF sPD1 levels, especially in active disease.^23,26–29^ There are very few data in the literature regarding sPD1 in JIA and they are centred in specific JIA subtypes. Lower serum sPD1 levels have been reported in sJIA compared to HCs and they were inversely correlated with the disease activity.^30^ On the other hand, Sag et al. did not find a significant difference in sPD1 levels between 24 JIA patients with oligoarthritis and HCs, nor an association with the disease activity. SF sPD1 levels were remarkably higher compared to the sera levels.^31^

In line with Sag’s study, our study, including all JIA subtypes, reports significantly higher SF sPD1 levels compared to the sera levels, without an association with the disease activity. Furthermore, no significant association of the sera sPD1 with JIA was detected. Therefore, we can speculate that sPD1 is mainly produced and acts at the site of the inflammation and that its peripheral levels do not directly reflect the ongoing inflammatory procedures at the joints. Moreover, contrary to previously reported observations, patients with sJIA in our study tended to have higher sPD1 levels compared to the other JIA subtypes, although the difference was not significant. Since sJIA is distinguished from the other JIA subtypes, due to its systemic rather than organ-specific phenotype and due to autoinflammatory as well as autoimmune characteristics, differences in the immunoregulatory profile of these patients could be expected.

Our study has certain limitations. It was not entirely prospective, since we included stored samples from our biobank. Although we recruited an adequate number of JIA patients, the number of paired samples from the same patients was relatively small. We performed a quantitative determination of the PD1 pathway’s components in JIA patients, but did not proceed to a qualitative assessment of the pathway’s functions. Despite these drawbacks, this is the first study in a Greek homogenous JIA population, investigating a novel and minimally studied pathway in paediatric patients with inflammatory arthritis. We consider our findings as an important addition to scientific data regarding the unravelling of the PD1 pathway in JIA.

In summary, our study showed an increased number of PD1-expressing CD4 and CD8 T-cells in the PB of JIA patients which correlated with the disease activity. At the site of inflammation, the above-mentioned cells were even higher as were the levels of sPD1. However, no association of serum sPD1 with disease characteristics or with the other components of the pathway was detected. Further investigation in a larger number of JIA patients is proposed to verify our observations and to demystify the precise role of the PD1 pathway in the pathogenesis and persistence of the joint inflammation in children and adolescents.

## NOTE

Preliminary results of this work have been previously presented and published as abstracts in scientific conferences [(Paediatric Rheumatology 2021;19 (Suppl 1):155, Ann Rheum Dis 2022;81(Suppl 1)].

## Figures and Tables

**Figure:**
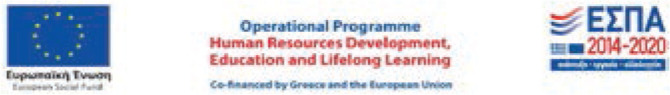


## ABBREVIATIONS

ANA: Antinuclear antibodies
CD: Cluster of differentiation
CXCR5: C-X-C chemokine receptor type 5
ELISA: Enzyme-linked immunosorbent assay
ERA: Enthesitis-related arthritis
ESR: Erythrocyte sedimentation rate
HCs: Healthy controls
ICs: Immune Checkpoints
ILAR: International League of Associations for Rheumatology
JADAS-10: Juvenile Arthritis Disease Activity Score-10
JIA: Juvenile Idiopathic Arthritis
MD-VAS: Physician’s global assessment on a visual analogue scale
PB: Peripheral blood
PD-1: Programmed cell Death protein-1
PDL-1 and 2: PD ligands 1 and 2
SF: Synovial fluid
Tregs: T-regulatory cells
anti-TNF: Anti-Tumour Necrosis Factor
hi: High
mRNA: Messenger Ribonucleic Acid
ns: Not significant
pg/ml: Picogram/millilitre
sJIA: Systemic JIA
sPD-1: Soluble PD-1
